# Academic stress and employment anxiety among graduating students: intolerance of uncertainty and future-oriented repetitive negative thinking as sequential cognitive-affective pathways

**DOI:** 10.3389/fpsyt.2026.1897109

**Published:** 2026-08-19

**Authors:** Mei Zhou, Na Ni

**Affiliations:** 1 School of Foreign Studies, Ankang University, Ankang, Shaanxi, China; 2 Faculty of Education, Universiti Teknologi MARA, Shah Alam, Malaysia

**Keywords:** academic stress, employment anxiety, future-oriented repetitive negative thinking, graduating students, intolerance of uncertainty, mental health

## Abstract

Graduating students often face the dual challenge of completing academic requirements and entering an uncertain labor market, yet limited research has examined the cognitive-affective factors involved in the association between academic stress and later employment anxiety. This study examined whether the association between academic stress and later employment anxiety included indirect associations through intolerance of uncertainty and future-oriented repetitive negative thinking in a theoretically specified sequential model. A three-wave timelagged survey was conducted among 1,045 graduating students in China. Academic stress, baseline employment anxiety, and covariates were measured at Time 1; intolerance of uncertainty and future-oriented repetitive negative thinking were measured at Time 2; and employment anxiety was measured at Time 3. Structural equation modeling indicated that Time 1 academic stress was positively associated with Time 3 employment anxiety after adjustment for baseline employment anxiety and demographic covariates. Time 1 academic stress was also positively associated with both Time 2 cognitive variables. Time 2 intolerance of uncertainty was positively associated with Time 3 employment anxiety and Time 2 future-oriented repetitive negative thinking, while Time 2 future-oriented repetitive negative thinking was positively associated with Time 3 employment anxiety. Bootstrap analyses identified significant indirect associations through intolerance of uncertainty, through future-oriented repetitive negative thinking, and through the theoretically specified sequential pathway involving both variables. Because the two intermediate variables were measured at the same wave, their ordering was theoretically specified rather than temporally established. The estimated total indirect association accounted for more than half of the total association between academic stress and later employment anxiety. These findings suggest that the association between academic stress and later employment anxiety may involve both uncertainty-related cognitive vulnerability and persistent future-oriented negative thinking. These factors may be relevant considerations for university mental health and career-support services.

## 1 Introduction

The transition from university to employment is a critical developmental period in which graduating students must complete academic requirements while preparing for uncertain labor-market entry. During this period, academic stress is not limited to examinations or coursework, but also includes thesis completion, internship performance, qualification examinations, peer comparison, and the expectation that academic achievement will translate into future career success. Such stressors may become closely connected with students’ psychological well-being, especially when academic performance is perceived as a signal of future employability. Prior research has shown that academic stress is associated with poorer mental health outcomes among students, including anxiety, depression, sleep problems, burnout, and reduced well-being (1–4). Recent evidence further suggests that academic pressure may have prospective implications for depressive symptoms and self-harm-related outcomes among young people (5). These findings highlight the need to understand academic stress not only as an educational issue but also as a mental health risk factor during key transition periods.

Employment anxiety is a particularly salient psychological concern among graduating students. It refers to worry, tension, and apprehension about job search, recruitment competition, career prospects, and the possibility of failing to obtain satisfactory employment. Existing studies have begun to examine the relationship between academic stress and employment anxiety. For example, recent research has shown that perceived academic stress is positively associated with employment anxiety among college students, with psychological resilience serving as a mediating factor (6). Related studies have also linked academic stress with academic burnout and reduced student well-being (4, 7), while career anxiety has been associated with students’ career goals and academic functioning (8). However, existing research has mainly emphasized general protective resources, such as resilience or support, whereas less is known about the cognitive-affective vulnerability processes through which academic stress is translated into employment anxiety during the graduation transition.

Intolerance of uncertainty may be one key mechanism linking academic stress to employment anxiety. It refers to the tendency to perceive uncertain situations as stressful, threatening, or difficult to tolerate (9). The education-to-employment transition is inherently uncertain: students may be unsure whether they can graduate smoothly, pass employment-related examinations, receive interview opportunities, meet employer expectations, or achieve stable career development. When academic stress is high, such uncertainty may become more salient and threatening. In this context, academic stress may increase students’ intolerance of uncertainty by making future academic and employment outcomes appear less predictable and less controllable. This mechanism is supported by prior evidence showing that intolerance of uncertainty is closely related to anxiety and depressive symptoms (10) and to future career anxiety and academic burnout among university students (11, 12).

Future-oriented repetitive negative thinking may further explain how uncertainty-related vulnerability develops into employment anxiety. Repetitive negative thinking is a perseverative and difficult-to-control pattern of thinking about negative content, and it has been widely discussed as a transdiagnostic process involved in anxiety and depressive symptoms (13–15). For graduating students, this process may take a future-oriented form, such as repeatedly thinking about failing to find a job, falling behind peers, making the wrong career choice, or being unable to cope with future occupational demands. Such thoughts are not merely normal career planning; rather, they represent persistent negative anticipation that may maintain and intensify anxiety. Intolerance of uncertainty may encourage this form of thinking because students who cannot tolerate uncertain outcomes may repeatedly attempt to mentally resolve future risks, but this repetitive thinking may instead amplify perceived threat and strengthen employment anxiety.

To address these gaps, the present study examines the prospective and indirect associations between academic stress and employment anxiety among graduating students. Specifically, we test whether the observed association between Time 1 academic stress and Time 3 employment anxiety includes indirect associations through Time 2 intolerance of uncertainty and future-oriented repetitive negative thinking. This study contributes to the literature in three ways. First, it situates employment anxiety within the broader mental health correlates of academic stress during the education-to-employment transition. Second, it extends prior work by moving beyond general protective-resource explanations and examining uncertainty-related and perseverative cognitive-affective vulnerability processes. Third, it uses a three-wave time-lagged design, measuring academic stress and baseline employment anxiety at Time 1, intolerance of uncertainty and future-oriented repetitive negative thinking at Time 2, and employment anxiety at Time 3. This design provides temporal separation between academic stress and subsequent employment anxiety and permits adjustment for baseline employment anxiety. Nevertheless, because the study is observational, the estimated prospective and indirect associations should not be interpreted as evidence that academic stress causes employment anxiety or changes in the proposed intermediate variables.

## 2 Literature review and hypotheses development

### 2.1 Academic stress and employment anxiety among graduating students

The transition from university to employment is a psychologically demanding period in which students are required to complete academic tasks while simultaneously preparing for an uncertain labor market. For graduating students, academic stress is not limited to examination pressure or coursework overload. It also includes thesis completion, internship requirements, professional qualification examinations, postgraduate entrance examinations, performance ranking, and the expectation that academic performance will translate into career opportunities. Prior studies have consistently shown that academic pressure is associated with poorer mental health outcomes among students, including psychological distress, depressive symptoms, anxiety, sleep problems, burnout, and reduced well-being (1–5). Recent longitudinal and review evidence further suggests that academic pressure is not merely a short-term educational burden but a risk factor that may contribute to later emotional difficulties and self-harm-related outcomes in young people (2, 5). Employment anxiety refers to a future-oriented state of worry, tension, and apprehension related to job search, occupational competition, career prospects, and the possibility of failing to obtain satisfactory employment. It is particularly salient among graduating students because employment outcomes are closely tied to academic achievement, perceived personal competence, social comparison, family expectations, and broader economic uncertainty. Recent research has begun to link academic stress with employment-related anxiety among university students. For example, studies on college students have shown that perceived academic stress is positively associated with employment anxiety and that psychological resources may partly explain this relationship (6). Research on career anxiety also suggests that uncertainty about career choice, future goals, and labor-market entry can undermine academic and psychological functioning (8).

From a cognitive appraisal perspective, academic stress may intensify employment anxiety because students interpret academic difficulties as signals of future career disadvantage. A graduating student who experiences repeated academic overload may not only feel exhausted in the present but may also infer that he or she is less competitive in the labor market. Similarly, difficulties in completing academic requirements may heighten concern about whether one can meet employer expectations, pass recruitment procedures, or achieve stable occupational development. Thus, academic stress may become psychologically linked to employment anxiety through the perceived continuity between academic performance and future career success.

Accordingly, the following hypothesis is proposed:

H1. Academic stress is positively associated with employment anxiety among graduating students.

### 2.2 Academic stress and intolerance of uncertainty

Although the direct relationship between academic stress and employment anxiety is important, a more theoretically informative question is why academic stress is transformed into employment-related anxiety. The present study argues that intolerance of uncertainty is a key cognitive vulnerability in this process. Intolerance of uncertainty refers to the tendency to perceive uncertain situations as stressful, threatening, or unacceptable, regardless of the actual probability of negative outcomes (16, 17). It reflects not only a dislike of ambiguity but also a broader cognitive disposition in which uncertain future events are interpreted as potentially dangerous and difficult to tolerate (9, 18). Graduating students are exposed to multiple forms of uncertainty. Academic outcomes, degree completion, examination performance, internship evaluations, recruitment results, and career trajectories are all uncertain. When academic stress is high, these uncertainties may become more salient and threatening. For example, a student under intense academic pressure may become more sensitive to ambiguous academic feedback, more worried about whether graduation requirements can be completed, and more likely to view unclear future outcomes as signs of potential failure. In this sense, academic stress may increase intolerance of uncertainty by making the future appear less predictable and less controllable.

This mechanism is especially relevant in the education-to-employment transition. Students do not experience academic demands in isolation; rather, academic performance is often perceived as a gateway to employment opportunities. When academic stress accumulates, students may become less tolerant of uncertain academic and career outcomes. Recent evidence has linked intolerance of uncertainty with negative academic and mental health outcomes among university students, including academic burnout and future career anxiety (11, 12). This suggests that intolerance of uncertainty can serve as a cognitive bridge between educational stressors and mental health risks.

Accordingly, the following hypothesis is proposed:

H2. Academic stress is positively associated with intolerance of uncertainty among graduating students.

### 2.3 Intolerance of uncertainty and employment anxiety

The job-search process is inherently uncertain. Graduating students often cannot fully predict whether they will receive interview opportunities, how employers will evaluate them, whether their qualifications will be sufficient, or whether available jobs will match their expectations. For students with high intolerance of uncertainty, this ambiguity may be experienced as a persistent threat. They may overestimate the negative implications of not knowing, interpret delayed responses from employers as signs of rejection, and perceive normal labor-market uncertainty as evidence of personal inadequacy. The broader clinical and psychological literature has identified intolerance of uncertainty as an important vulnerability factor for anxiety and related emotional difficulties. Meta-analytic and empirical evidence indicates that intolerance of uncertainty is associated with symptoms of generalized anxiety, depression, obsessive–compulsive symptoms, and other anxiety-related problems (10, 19). Contemporary models further suggest that fear of the unknown is a central component of anxiety because uncertain future threats are difficult to predict, control, or dismiss (9). In the employment context, uncertainty is not abstract; it is tied to concrete concerns about job availability, income, social comparison, family expectations, and long-term career identity.

For graduating students, intolerance of uncertainty may therefore directly increase employment anxiety. Students who are less able to tolerate uncertain outcomes may engage in excessive monitoring of recruitment information, repeatedly compare themselves with peers, avoid job-search activities because they fear failure, or experience persistent anticipatory anxiety before interviews and examinations. Empirical evidence among Chinese undergraduates also suggests that intolerance of uncertainty is associated with future career anxiety and related emotional difficulties (11). Therefore, intolerance of uncertainty is expected to be a proximal cognitive risk factor for employment anxiety. Furthermore, intolerance of uncertainty may explain why academic stress is associated with employment anxiety. Academic stress may first heighten students’ perception that future academic and career outcomes are unpredictable; this intolerance of uncertainty may then increase employment-related worry and fear. In this sense, intolerance of uncertainty functions as a mediating cognitive pathway between academic stress and employment anxiety.

Accordingly, the following hypotheses are proposed:

H3. Intolerance of uncertainty is positively associated with employment anxiety among graduating students.
H4. Intolerance of uncertainty mediates the relationship between academic stress and employment anxiety.

### 2.4 Future-oriented repetitive negative thinking as a cognitive-affective pathway

Future-oriented repetitive negative thinking is proposed as the second key pathway in the present model. Repetitive negative thinking refers to a perseverative, difficult-to-control pattern of thinking about negative content, and it has been conceptualized as a transdiagnostic process across anxiety and depressive disorders (13, 14, 20, 21). Although repetitive negative thinking may take different forms, such as worry and rumination, its common features include repetitiveness, perceived uncontrollability, negative valence, and difficulty disengaging from distressing thoughts (13, 21). Recent meta-analytic evidence also indicates that reducing repetitive negative thinking is relevant to the treatment of youth depression and anxiety (15). In the present study, future-oriented repetitive negative thinking refers to persistent and difficult-to-control negative thoughts about future academic and employment outcomes. For graduating students, this may include repeated thoughts such as “What if I cannot find a job?”, “What if my academic record is not competitive?”, “What if I fail the interview?”, or “What if my future career development is worse than that of my peers?” These thoughts are not merely normal career planning. Rather, they represent a cognitive-affective process in which students repeatedly simulate negative future scenarios without reaching a constructive solution.

Intolerance of uncertainty is likely to increase future-oriented repetitive negative thinking. When students cannot tolerate uncertain outcomes, they may attempt to mentally reduce uncertainty by repeatedly thinking about possible future events. However, such thinking often fails to produce certainty. Instead, it may generate more negative possibilities, increase perceived threat, and reinforce anxious anticipation. This is consistent with research indicating that repetitive negative thinking is closely linked to worry, rumination, anxiety, and depressive symptoms (13, 14, 22). Therefore, students with higher intolerance of uncertainty may be more likely to engage in future-oriented repetitive negative thinking during the transition from university to employment. Future-oriented repetitive negative thinking is also expected to increase employment anxiety. Repeated negative anticipation may amplify the perceived probability and cost of employment failure. It may also maintain attention on threatening career-related information, reduce confidence in coping with job-search challenges, and make employment uncertainty feel more urgent and uncontrollable. As a result, graduating students who repeatedly think about negative future employment outcomes are likely to experience stronger employment anxiety.

Accordingly, the following hypotheses are proposed:

H5. Intolerance of uncertainty is positively associated with future-oriented repetitive negative thinking.
H6. Future-oriented repetitive negative thinking is positively associated with employment anxiety.

### 2.5 Sequential cognitive-affective pathway from academic stress to employment anxiety

Building on the above arguments, the present study proposes a sequential cognitive-affective pathway linking academic stress to employment anxiety. Academic stress may first increase intolerance of uncertainty because academic demands make future outcomes feel more ambiguous, uncontrollable, and threatening. This heightened intolerance of uncertainty may then trigger future-oriented repetitive negative thinking, as students repeatedly attempt to anticipate, explain, or mentally control uncertain future outcomes. However, instead of resolving uncertainty, this repetitive negative thinking may maintain and intensify employment-related anxiety. This sequential pathway extends previous research in several ways. First, whereas prior work has mainly emphasized protective resources such as psychological resilience in the relationship between academic stress and employment anxiety (6), the present study focuses on a cognitive-affective vulnerability process. Second, by integrating intolerance of uncertainty and repetitive negative thinking, the model explains not only whether academic stress is associated with employment anxiety but also how academic stress may be transformed into persistent future-oriented anxiety. Third, the model situates employment anxiety within a broader mental health framework, because both intolerance of uncertainty and repetitive negative thinking have been widely linked to anxiety, depression, and related psychological difficulties (10, 13–15, 19).

The proposed pathway is particularly meaningful for graduating students. During the education-to-employment transition, academic stress and career uncertainty are closely intertwined. A student who experiences academic overload may begin to perceive future academic and employment outcomes as uncertain and threatening. If the student is unable to tolerate this uncertainty, he or she may repeatedly imagine negative future scenarios, such as failing to graduate smoothly, being rejected by employers, or falling behind peers. This future-oriented repetitive negative thinking may then intensify employment anxiety. Therefore, the present study conceptualizes intolerance of uncertainty and future-oriented repetitive negative thinking as sequential cognitive-affective pathways between academic stress and employment anxiety.

Accordingly, the following hypothesis is proposed:

H7. Academic stress is indirectly associated with employment anxiety through a theoretically specified sequential pathway involving intolerance of uncertainty and future-oriented repetitive negative thinking.

### 2.6 Research model and hypotheses

**Figure 1:**
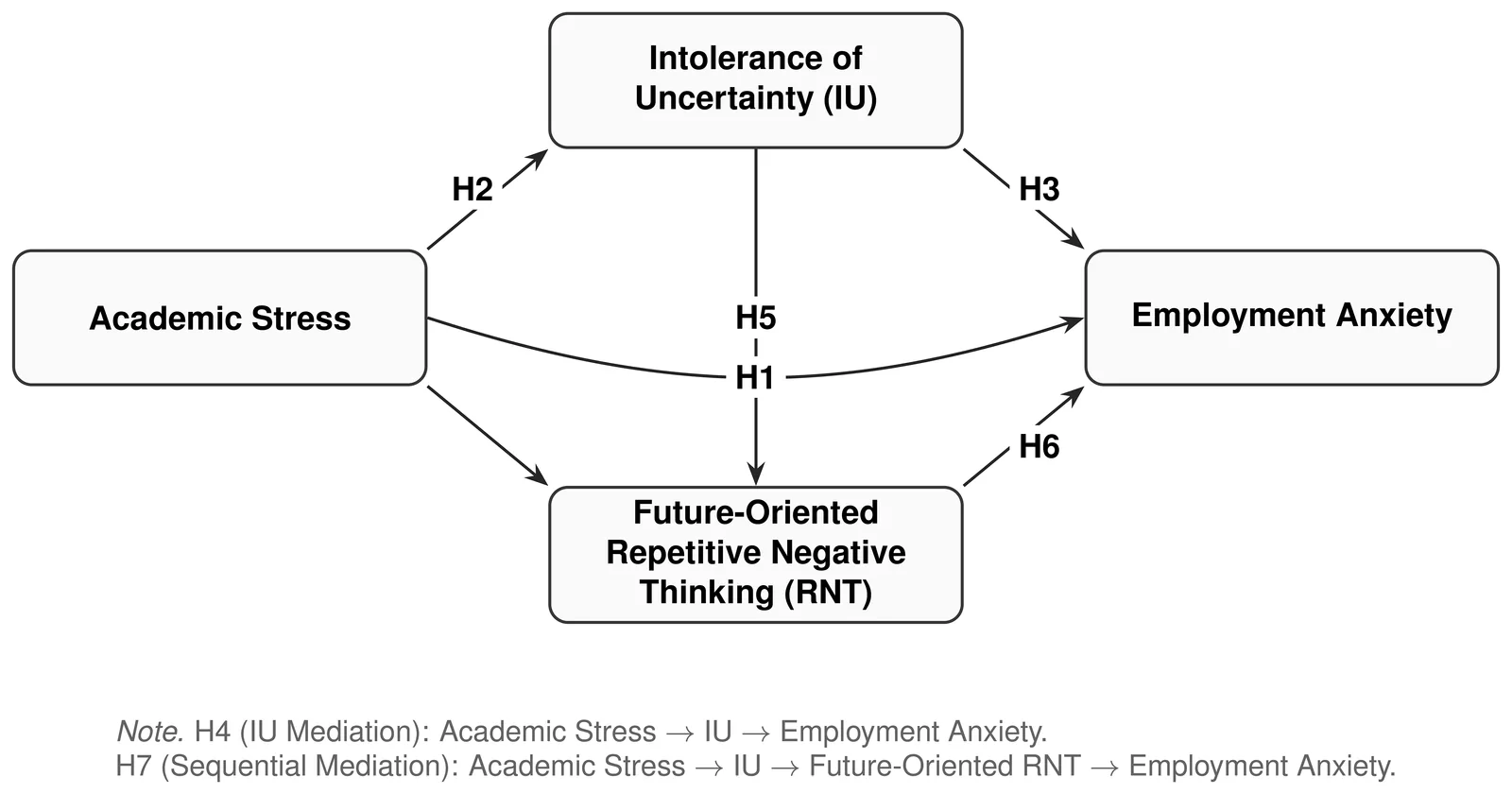
The hypothesized sequential mediation model. H4 and H7 represent individual and sequential mediation pathways, respectively, and are determined by combinations of the depicted structural paths.

Based on the above literature review, this study develops a theoretically specified sequential mediation model in which academic stress is associated with employment anxiety through intolerance of uncertainty and future-oriented repetitive negative thinking (**Figure 1**). The directional ordering from intolerance of uncertainty to future-oriented repetitive negative thinking is based on the theoretical proposition that difficulty tolerating uncertainty may be associated with persistent attempts to anticipate and mentally resolve future threats. However, because both mediators were measured at Time 2, the model does not establish temporal precedence between them. Accordingly, the sequential indirect effect is interpreted as a statistical indirect association consistent with the proposed theoretical ordering rather than as evidence of a temporally demonstrated sequence.

The hypotheses of the present study are summarized as follows:

H1. Academic stress is positively associated with employment anxiety among graduating students.
H2. Academic stress is positively associated with intolerance of uncertainty among graduating students.
H3. Intolerance of uncertainty is positively associated with employment anxiety among graduating students.
H4. Intolerance of uncertainty mediates the relationship between academic stress and employment anxiety.
H5. Intolerance of uncertainty is positively associated with future-oriented repetitive negative thinking.
H6. Future-oriented repetitive negative thinking is positively associated with employment anxiety.
H7. Academic stress is indirectly associated with employment anxiety through a theoretically specified sequential pathway involving intolerance of uncertainty and future-oriented repetitive negative thinking.

## 3 Materials and methods

### 3.1 Study design and participants

This study adopted a three-wave time-lagged survey design to examine the sequential cognitive-affective pathway from academic stress to employment anxiety among graduating students. Data were collected from full-time graduating students at participating universities in Shaanxi Province, China, during the autumn semester of 2025. Time 1 data were collected from September 15 to September 30, Time 2 data from October 15 to October 31, and Time 3 data from November 15 to November 30, with an interval of approximately four weeks between adjacent waves. At Time 1, academic stress, baseline employment anxiety, and demographic and job-search-related covariates were measured. At Time 2, intolerance of uncertainty and future-oriented repetitive negative thinking were assessed. At Time 3, employment anxiety was measured as the main outcome. This design separated the predictor, mediators, and outcome across waves while controlling for baseline employment anxiety.

This study used a multisite, non-probability convenience sampling strategy. The participating universities were identified through the research team’s existing institutional contacts and their willingness to facilitate recruitment. Within the participating universities, trained research assistants contacted accessible final-year undergraduate and graduating master’s student classes and distributed recruitment announcements through class-based communication channels and online student groups. The universities, classes, and individual students were not selected through probability-based random sampling. The classes served as channels for distributing recruitment information rather than as randomly selected sampling clusters.

Participants were eligible if they were aged 18 years or above, enrolled as final-year undergraduate students or graduating master’s students, able to complete a Chinese-language questionnaire, and preparing for employment, internship, postgraduate entrance examinations, civil-service examinations, teacher recruitment examinations, or other post-graduation transitions. Responses were excluded for failed attention checks, duplicate submissions, inconsistent self-generated identification codes, unrealistically short completion time, or straight-line response patterns. A total of 1,518 students were invited to participate. Among them, 1,376 completed the Time 1 questionnaire, 1,229 completed Time 2, and 1,084 completed Time 3. After excluding 39 invalid matched responses, the final analytic sample consisted of 1,045 graduating students.

### 3.2 Procedure and ethical considerations

Participants were recruited through class-based announcements and online notices distributed by trained research assistants. Before accessing the questionnaire, all participants read an electronic informed consent form that explained the study purpose, survey procedure, voluntary nature of participation, confidentiality protection, and right to withdraw. Only students who selected the consent option were allowed to proceed to the questionnaire. To match responses across the three waves while preserving anonymity, participants generated a unique identification code at each wave. The code consisted of the first two letters of a self-chosen memorable word, the participant’s birth month, and a two-digit self-chosen number. No names, student numbers, telephone numbers, national identification numbers, or other directly identifying information were collected. The studies involving human participants were reviewed and approved by the Ethics Committee of the School of Foreign Studies, Ankang University (Approval No. AKWY-202518). The research was conducted in accordance with the Declaration of Helsinki. All participants provided electronic informed consent before participation. The dataset was stored on a password-protected computer accessible only to the research team, and all analyses were conducted using anonymized data.

### 3.3 Measures

All measures were administered in Chinese. The instruments differed in their language origin and adaptation procedures. The College Graduates’ Employment Anxiety Questionnaire was originally developed in Chinese for Chinese university graduates and therefore required no translation. Previously validated Chinese item wording was used for the Intolerance of Uncertainty Scale–12 and the Perseverative Thinking Questionnaire (23, 24). The Perception of Academic Stress Scale was translated from English into Chinese for the present study using a forward-translation and back-translation procedure. For the Perception of Academic Stress Scale, a bilingual psychology researcher first translated the original English items into Chinese. A second bilingual researcher, who had not reviewed the original English items, independently translated the Chinese version back into English. The research team compared the back-translated version with the original instrument and resolved discrepancies through discussion, with emphasis on semantic and conceptual equivalence in the Chinese university context. For the Perseverative Thinking Questionnaire, the validated Chinese item wording was retained, while the instructions were contextualized to ask participants to consider negative thinking related to academic completion, future employment, and post-graduation life during the previous two weeks. Two bilingual psychology researchers reviewed the contextualized instructions to ensure that the adaptation changed the situational focus without altering the content or meaning of the original items.

#### 3.3.1 Academic stress

Academic stress was measured at Time 1 using the 18-item Perception of Academic Stress Scale developed by Bedewy and Gabriel (25). The scale assesses perceived academic expectations, workload and examinations, academic self-perceptions, and time restraints. In the original development study, the total scale demonstrated acceptable internal consistency (Cronbach’s *α* = 0.70), evidence of content validity, and a four-factor structure comprising theoretically meaningful dimensions. As described above, the scale was translated into Chinese for the present study using forward translation, independent back-translation, and comparison with the original English version. Items were rated on a 5-point Likert scale ranging from 1 = strongly disagree to 5 = strongly agree. Positively worded items were reverse-coded so that higher scores indicated higher perceived academic stress. The mean score of all items was used. In the present sample, Cronbach’s *α* was 0.87 and McDonald’s *ω* was 0.88.

#### 3.3.2 Intolerance of uncertainty

Intolerance of uncertainty was measured at Time 2 using the 12-item short form of the Intolerance of Uncertainty Scale (26). The scale assesses the extent to which individuals perceive uncertain situations as stressful, unacceptable, and difficult to tolerate. The original IUS–12 demonstrated excellent internal consistency for the total scale (Cronbach’s *α* = 0.91) and good internal consistency for its two dimensions (both *α* = 0.85). Confirmatory factor analyses supported a stable two-factor structure, and the IUS–12 correlated strongly with the original 27-item scale (*r* = 0.96) and significantly with established measures of worry and anxiety, supporting convergent validity. The present study used previously validated Chinese item wording; prior validation in Chinese-speaking adult samples supported the construct validity of the Chinese IUS–12 (23). Items were rated on a 5-point Likert scale ranging from 1 = not at all characteristic of me to 5 = entirely characteristic of me. The mean score was calculated, with higher scores indicating greater intolerance of uncertainty. In the present sample, Cronbach’s *α* and McDonald’s *ω* were both 0.84.

#### 3.3.3 Future-oriented repetitive negative thinking

Future-oriented repetitive negative thinking was measured at Time 2 using the 15-item Perseverative Thinking Questionnaire developed by Ehring et al. (21). The PTQ assesses repetitive, intrusive, and difficult-to-disengage negative thinking, together with its perceived unproductiveness and occupation of mental capacity. In the original validation studies, the PTQ total score demonstrated excellent internal consistency across samples (Cronbach’s *α* = 0.94–0.95) and satisfactory four-week test–retest reliability (*r* = 0.69). Its second-order factor structure was supported across clinical and non-clinical samples, and substantial correlations with established measures of worry and rumination supported convergent validity. The present study used validated Chinese item wording (24). The items themselves were not rewritten; only the instructions were contextualized to ask participants to respond with reference to thoughts about academic completion, future employment, and post-graduation life during the previous two weeks. Items were rated on a 5-point scale ranging from 0 = never to 4 = almost always. The mean score was used, with higher scores indicating more frequent future-oriented repetitive negative thinking. In the present sample, Cronbach’s *α* was 0.91 and McDonald’s *ω* was 0.92.

#### 3.3.4 Employment anxiety

Employment anxiety was measured at Time 1 and Time 3 using the 26-item College Graduates’ Employment Anxiety Questionnaire developed in Chinese by Zhang and Chen (27). The questionnaire was specifically designed for Chinese university graduates and therefore required no translation. It comprises four dimensions: employment competition pressure, lack of employment support, lack of self-confidence, and concerns about employment prospects. The original scale demonstrated an internal consistency coefficient of 0.71 and test–retest reliability of 0.79, and subsequent cross-regional research supported the stability of its four-factor structure (28). The questionnaire has also been used in recent research on employment anxiety among Chinese university students (6). Items were rated on a 5-point Likert scale ranging from 1 = strongly inconsistent with me to 5 = strongly consistent with me. Higher mean scores indicated higher levels of employment anxiety. Time 3 employment anxiety was the main outcome variable, while Time 1 employment anxiety was included as a baseline covariate in the primary structural model.

Wave-specific reliability was evaluated separately for employment anxiety because Time 1 employment anxiety was included as a baseline covariate, whereas Time 3 employment anxiety was specified as the primary outcome. The 26-item scale demonstrated good internal consistency at Time 1 (Cronbach’s *α* = 0.86, McDonald’s *ω* = 0.88) and at Time 3 (Cronbach’s *α* = 0.89, McDonald’s *ω* = 0.90).

#### 3.3.5 Control variables

The following control variables were measured at Time 1: gender, age, educational level, academic discipline, household registration, only-child status, monthly household income, internship experience, current job-search status, and perceived family employment expectation. Gender was coded as 0 = male and 1 = female. Educational level was coded as 0 = undergraduate student and 1 = master’s student. Academic discipline was classified into humanities and social sciences, economics and management, science and engineering, education, and medical and health-related disciplines. Household registration was coded as 0 = rural and 1 = urban. Only-child status was coded as 0 = no and 1 = yes. Monthly household income was divided into three categories: below 5,000 RMB, 5,000–10,000 RMB, and above 10,000 RMB. Internship experience was coded as 0 = no and 1 = yes. Current job-search status was coded as 0 = not yet started, 1 = preparing, 2 = actively applying, and 3 = received at least one offer. Perceived family employment expectation was measured using one item rated from 1 = very low to 5 = very high.

### 3.4 Data quality control and missing data

Several procedures were used to ensure data quality. First, each questionnaire included two attention check items, and participants who failed either attention check were excluded. Second, responses submitted in less than one-third of the median completion time for each wave were removed. Third, straight-line responses were identified when a participant selected the same response option for more than 90% of scale items within a wave. Fourth, duplicate submissions were screened using the survey platform record and the self-generated identification code, and only the earliest complete response was retained. Fifth, responses with inconsistent self-generated identification codes across waves were excluded from the matched dataset. All retained questionnaires had complete responses because scale items were set as mandatory after informed consent was provided. Therefore, no item-level missing data were present in the final analytic sample. Attrition was evaluated by comparing participants who completed all three waves with those who were lost to follow-up on Time 1 demographic characteristics, academic stress, and baseline employment anxiety. The final matched sample of 1,045 participants was used for all main analyses.

### 3.5 Statistical analysis

Data analyses were conducted using IBM SPSS Statistics 27.0 and R 4.4.2. Structural equation modeling was performed with the lavaan package in R (29). Descriptive statistics, Pearson correlations, reliability coefficients, and confirmatory factor analysis were first conducted. Reliability was evaluated using Cronbach’s alpha, McDonald’s omega, and composite reliability. Construct validity was assessed through a four-factor measurement model including academic stress, intolerance of uncertainty, future-oriented repetitive negative thinking, and employment anxiety.

Parcel-based indicators were constructed before estimation of the structural paths and without reference to the outcome associations or indirect-effect results. Academic stress was represented by four parcels corresponding to its theoretical dimensions of academic expectations, workload and examinations, academic self-perceptions, and time restraints. Employment anxiety was represented by four parcels corresponding to employment competition pressure, lack of employment support, lack of self-confidence, and concerns about employment prospects. Future-oriented repetitive negative thinking was represented by three content-based parcels corresponding to the core characteristics of repetitive negative thinking, perceived unproductiveness, and occupation of mental capacity. For the IUS–12, three four-item parcels were constructed using an item-to-construct balance procedure. The 12 items were ranked according to their standardized loadings in a preliminary single-factor model and distributed across the three parcels so that relatively high- and low-loading items were balanced within each parcel. This procedure resulted in 14 indicators across the four latent constructs. Parceling was used to reduce the number of freely estimated parameters while preserving the theoretical content of the multidimensional measures (30). Because parceling may conceal item-level misspecification, an additional four-factor confirmatory factor analysis using all 71 individual items was conducted as a sensitivity analysis. Model fit was evaluated using *χ*^2^*/df*, CFI, TLI, RMSEA, and SRMR. Values of CFI and TLI above 0.90 and RMSEA and SRMR below 0.08 were considered acceptable, while CFI and TLI above 0.95 and RMSEA and SRMR below 0.06 indicated good fit (31). Convergent validity was assessed using standardized factor loadings, composite reliability, and average variance extracted, and discriminant validity was evaluated using the Fornell–Larcker criterion (32).

Common method variance was examined by comparing the hypothesized four-factor model with a one-factor model and by estimating a common latent method factor model (33). The hypothesized sequential mediation model specified Time 1 academic stress as the independent variable, Time 2 intolerance of uncertainty and future-oriented repetitive negative thinking as mediators, and Time 3 employment anxiety as the outcome. Baseline employment anxiety and all control variables were included as covariates. Because the two mediators were measured at the same wave, their ordering was specified according to the theoretical model rather than temporal separation within Time 2. Accordingly, the sequential indirect effect was interpreted as a theory-based product-of-coefficients effect rather than evidence of temporal precedence between the two mediators. Indirect effects were tested using 5,000 bootstrap resamples with 95% confidence intervals. The tested effects included the indirect pathway through intolerance of uncertainty, the indirect pathway through future-oriented repetitive negative thinking, and the sequential pathway through both mediators. Robustness analyses re-estimated the model using observed scale mean scores, repeated the model without baseline employment anxiety, and conducted multi-group analyses by gender and educational level. Statistical significance was evaluated using two-tailed tests with *α* = 0.05.

For the multi-group analyses, measurement invariance was evaluated separately across gender and educational-level groups using the same parcel-based four-factor measurement model as in the primary analysis. Configural invariance specified the same factor structure across groups, metric invariance additionally constrained the factor loadings to equality, and scalar invariance further constrained the indicator intercepts to equality. Measurement invariance was considered supported when |δCFI| ≤ 0.010, δRMSEA ≤ 0.015, and δSRMR ≤ 0.030 for metric invariance or δSRMR ≤ 0.010 for scalar invariance (34). After measurement invariance had been established, the six primary structural paths were compared between groups using a metric-invariant measurement model. The unconstrained structural model allowed these paths to vary across groups, whereas the constrained model held the six paths equal across groups. Because the comparisons concerned structural regression paths rather than latent means, no latent mean comparisons were performed. Time 1 baseline employment anxiety and the remaining applicable covariates were retained in the structural comparison models. Gender was excluded as a covariate in the gender-based analysis, and educational level was excluded as a covariate in the educational-level analysis because the corresponding grouping variable had no within-group variation.

## 4 Results

### 4.1 Participant characteristics and attrition analysis

A total of 1,045 graduating students were included in the final analytic sample. As shown in **Table 1**, 452 participants were male (43.25%) and 593 were female (56.75%). The sample mainly consisted of final-year undergraduate students (85.26%), while graduating master’s students accounted for 14.74%. Participants came from diverse academic disciplines, with science and engineering representing the largest group (29.85%), followed by humanities and social sciences (22.39%), economics and management (20.86%), education (15.79%), and medical and health-related disciplines (11.11%). In terms of job-search status, most participants were either preparing for employment-related activities (39.43%) or actively applying for jobs (35.22%), indicating that the sample was well aligned with the education-to-employment transition context. Attrition analysis was conducted to compare participants retained in the final analytic sample with those who completed the Time 1 survey but were not included in the final matched sample. As shown in **Table 2**, there were no significant differences between retained and non-retained participants in age, gender, educational level, academic discipline distribution, Time 1 academic stress, or Time 1 employment anxiety. These results suggest that sample attrition did not introduce substantial systematic bias into the final analytic sample.

**Table 1:** Participant characteristics of the final analytic sample.

Characteristic	*n*	%
Gender		
Male	452	43.25
Female	593	56.75
Educational level		
Final-year undergraduate student	891	85.26
Graduating master’s student	154	14.74
Academic discipline		
Humanities and social sciences	234	22.39
Economics and management	218	20.86
Science and engineering	312	29.85
Education	165	15.79
Medical and health-related disciplines	116	11.11
Household registration		
Rural	612	58.56
Urban	433	41.44
Only-child status		
No	647	61.91
Yes	398	38.09
Monthly household income		
Below 5,000 RMB	489	46.79
5,000–10,000 RMB	391	37.42
Above 10,000 RMB	165	15.79
Internship experience		
No	321	30.72
Yes	724	69.28
Current job-search status		
Not yet started	143	13.68
Preparing	412	39.43
Actively applying	368	35.22
Received at least one offer	122	11.67

**Table 2:** Attrition analysis between retained and non-retained participants.

Variable	Retained (*n* = 1,045)	Non-retained (*n* = 331)	Test statistic	*p*
Age (*M* ± *SD*)	22.46 ± 1.18	22.49 ± 1.21	*t* =−0.40	0.689
Gender (Male, %)	43.25%	41.09%	*χ*^2^ = 0.40	0.528
Educational level (Undergrad, %)	85.26%	86.71%	*χ*^2^ = 0.32	0.574
Academic discipline distribution	–	–	*χ*^2^ = 0.26	0.992
Time 1 academic stress (*M* ± *SD*)	3.24 ± 0.68	3.21 ± 0.71	*t* = 0.69	0.489
Time 1 employment anxiety (*M* ± *SD*)	3.42 ± 0.62	3.45 ± 0.65	*t* =−0.76	0.448

### 4.2 Descriptive statistics and correlations

Descriptive statistics and Pearson correlations among the main study variables are presented in **Table 3**. The skewness values ranged from -0.27 to 0.32, and the kurtosis values ranged from -0.41 to 0.15, indicating no serious deviation from normality. Time 1 academic stress was positively correlated with Time 3 employment anxiety (*r* = 0.14, *p <* 0.001), providing preliminary support for the expected association between academic stress and subsequent employment anxiety. Time 1 academic stress was also positively correlated with Time 2 intolerance of uncertainty (*r* = 0.26, *p <* 0.001) and Time 2 future-oriented repetitive negative thinking (*r* = 0.07, *p <* 0.05). Time 2 intolerance of uncertainty was positively associated with Time 2 future-oriented repetitive negative thinking (*r* = 0.45, *p <* 0.001) and Time 3 employment anxiety (*r* = 0.28, *p <* 0.001). In addition, Time 2 future-oriented repetitive negative thinking was positively associated with Time 3 employment anxiety (*r* = 0.32, *p <* 0.001). These correlations were consistent with the hypothesized cognitive-affective pathway from academic stress to employment anxiety. Baseline employment anxiety was moderately correlated with Time 3 employment anxiety (*r* = 0.54, *p <* 0.001), indicating temporal stability in employment anxiety and supporting its inclusion as a baseline covariate in the subsequent structural equation model. As shown in **Table 4**, all four constructs demonstrated satisfactory internal consistency and composite reliability. The average variance extracted values exceeded 0.50, providing support for convergent validity.

**Table 3:** Descriptive statistics and correlations among main study variables.

Variable	*M*	*SD*	Skewness	Kurtosis	1	2	3	4	5
1. Time 1 academic stress	3.24	0.68	0.21	-0.34	–				
2. Time 1 employment anxiety	3.42	0.62	-0.14	0.08	0.33^∗∗∗^	–			
3. Time 2 intolerance of uncertainty	3.15	0.72	0.32	-0.12	0.26^∗∗∗^	0.09^∗∗^	–		
4. Time 2 future-oriented RNT	2.28	0.78	0.18	-0.41	0.07^∗^	0.18^∗∗∗^	0.45^∗∗∗^	–	
5. Time 3 employment anxiety	3.51	0.64	-0.27	0.15	0.14^∗∗∗^	0.54^∗∗∗^	0.28^∗∗∗^	0.32^∗∗∗^ –	

*N* = 1,045. RNT, repetitive negative thinking. Correlation coefficients are Pearson’s *r*. ^∗^*p <* 0.05, ^∗∗^*p <* 0.01, ^∗∗∗^*p <* 0.001.

**Table 4:** Reliability, convergent validity, and discriminant validity.

Construct	Cronbach’s *α*	McDonald’s *ω*	CR	AVE	Range of loadings
Academic stress	0.87	0.88	0.88	0.51	0.53–0.79
Intolerance of uncertainty	0.84	0.84	0.85	0.53	0.58–0.81
Future-oriented RNT	0.91	0.92	0.92	0.56	0.61–0.84
Time 3 employment anxiety	0.89	0.90	0.90	0.52	0.55–0.82

CR, composite reliability; AVE, average variance extracted; RNT, repetitive negative thinking.

### 4.3 Common method variance

Common method variance was examined using both model comparison and a common latent method factor approach. First, the one-factor measurement model showed poor fit to the data, with *χ*^2^ = 1845.29, *df* = 81, CFI = 0.483, TLI = 0.421, RMSEA = 0.144, and SRMR = 0.127 (**Table 5**). This result indicated that the variance in the measurement indicators could not be adequately explained by a single general factor. Second, a common latent method factor was added to the hypothesized four-factor measurement model. The inclusion of the common method factor only marginally improved model fit, and the changes in the key fit indices were small (δCFI = 0.012 and δRMSEA = 0.005). In addition, the standardized factor loadings and the substantive pattern of associations among the latent constructs remained stable after the method factor was included. Therefore, common method variance was unlikely to account for the main findings of the study.

**Table 5:** Confirmatory factor analysis and model comparisons.

Model	*χ*2	*df*	*χ* ^2^ */df*	CFI	TLI	RMSEA/SRMR
Parcel-based four-factor model	168.42	75	2.25	0.973	0.967	0.034/0.029
Item-level four-factor model	4411.46	2408	1.83	0.926	0.921	0.029/0.043
Three-factor model*^a^*	542.18	78	6.95	0.864	0.841	0.075/0.068
Two-factor model*^b^*	987.63	80	12.35	0.734	0.698	0.104/0.092
One-factor model*^c^*	1845.29	81	22.78	0.483	0.421	0.144/0.127

The parcel-based four-factor model used 14 indicators, whereas the item-level four-factor model used all 71 individual items. The three-factor, two-factor, and one-factor models were estimated using the parcel-based indicators.

a Combined intolerance of uncertainty and future-oriented RNT into a single factor.

b Combined academic stress, intolerance of uncertainty, and future-oriented RNT into a single factor.

c All indicators loaded onto a single general factor.

As an additional sensitivity analysis, we estimated a four-factor measurement model using all 71 individual items rather than parcel-based indicators. The item-level model demonstrated acceptable fit to the data: *χ*^2^ = 4411.46, *df* = 2408, *χ*^2^*/df* = 1.83, CFI = 0.926, TLI = 0.921, RMSEA = 0.029, and SRMR = 0.043.

Although the incremental fit indices were lower than those of the parcel-based model, all indices met the prespecified criteria for acceptable fit. These results support the four-factor measurement structure at the item level and indicate that the adequacy of the measurement model was not dependent solely on parcel construction.

### 4.4 Testing of the hypothesized structural model

**Figure 2:**
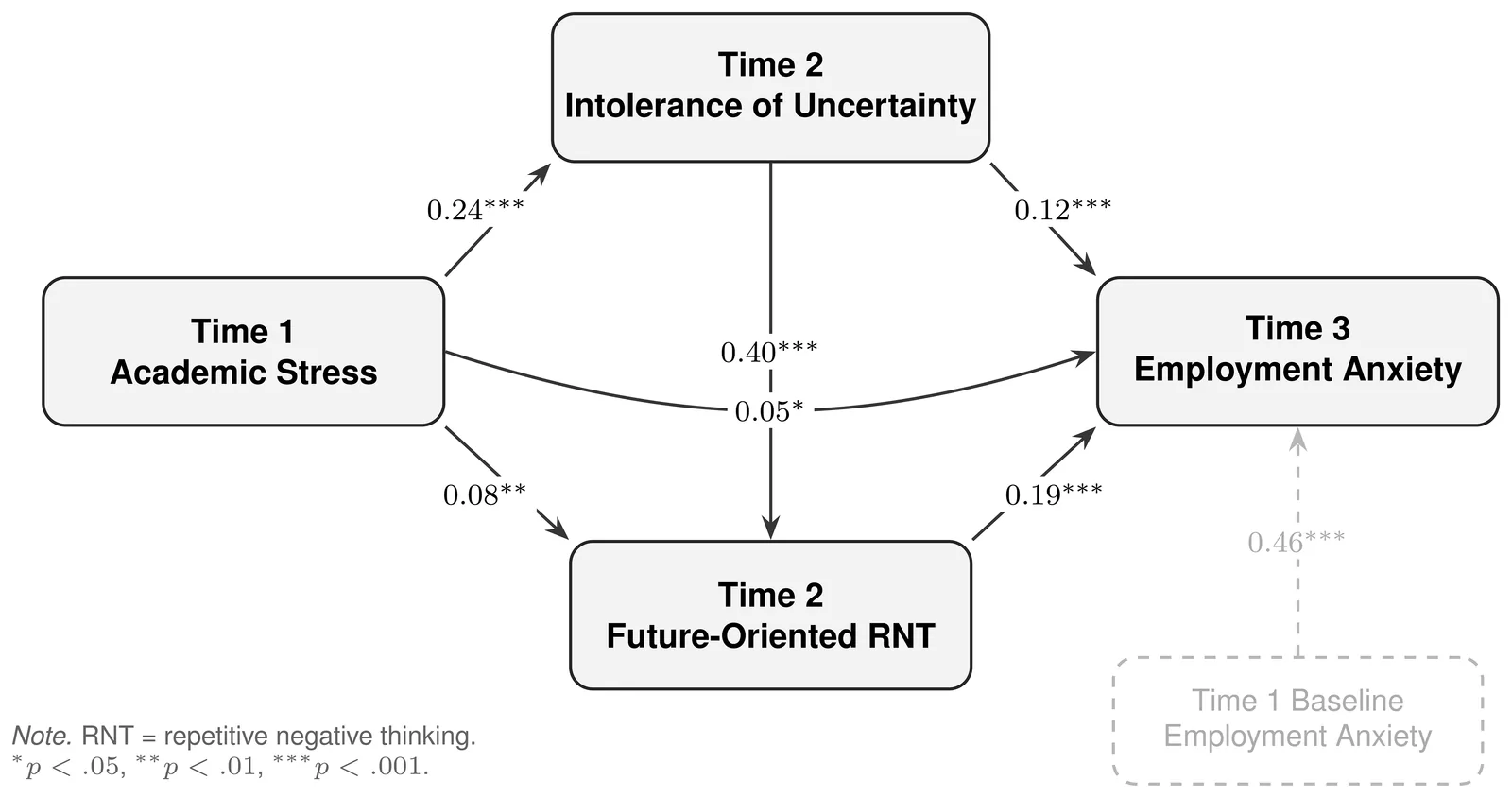
Standardized results of the structural equation model. Path coefficients represent standardized estimates (*β*). Baseline employment anxiety and demographic covariates were controlled for in the model; demographic covariates are not displayed for clarity. The ordering from intolerance of uncertainty to future-oriented repetitive negative thinking reflects the hypothesized cognitive-affective pathway.

The hypothesized structural equation model demonstrated good fit to the data: *χ*^2^ = 193.65, *df* = 84, *χ*^2^*/df* = 2.31, CFI = 0.966, TLI = 0.958, RMSEA = 0.035, and SRMR = 0.033. The standardized path coefficients are reported in **Table 6**, and the structural model is illustrated in **Figure 2**.

**Table 6:** Standardized path coefficients of the structural model.

Path	*β*	*SE*	95% CI	*p*
Time 1 academic stress → Time 3 employment anxiety	0.05	0.02	[0.01, 0.09]	0.023
Time 1 academic stress → Time 2 intolerance of uncertainty	0.24	0.03	[0.18, 0.30]	*<* 0.001
Time 1 academic stress → Time 2 future-oriented RNT	0.08	0.03	[0.02, 0.14]	0.008
Time 2 intolerance of uncertainty → Time 3 employment anxiety	0.12	0.03	[0.06, 0.18]	*<* 0.001
Time 2 intolerance of uncertainty → Time 2 future-oriented RNT	0.40	0.03	[0.34, 0.46]	*<* 0.001
Time 2 future-oriented RNT → Time 3 employment anxiety	0.19	0.03	[0.13, 0.25]	*<* 0.001
Time 1 baseline employment anxiety → Time 3 employment anxiety	0.46	0.03	[0.40, 0.52]	*<* 0.001

Standardized coefficients are reported. RNT, repetitive negative thinking; CI, confidence interval. Baseline employment anxiety and all control variables were included in the model.

After controlling for baseline employment anxiety and demographic covariates, Time 1 academic stress remained positively associated with Time 3 employment anxiety (*β* = 0.05, *SE* = 0.02, 95% CI [0.01, 0.09], *p* = 0.023), supporting H1. Time 1 academic stress was also positively associated with Time 2 intolerance of uncertainty (*β* = 0.24, *SE* = 0.03, 95% CI [0.18, 0.30], *p <* 0.001), supporting H2. In addition, Time 1 academic stress was positively associated with Time 2 future-oriented repetitive negative thinking (*β* = 0.08, *SE* = 0.03, 95% CI [0.02, 0.14], *p* = 0.008), indicating a small but statistically significant association. Time 2 intolerance of uncertainty was positively associated with Time 3 employment anxiety (*β* = 0.12, *SE* = 0.03, 95% CI [0.06, 0.18], *p <* 0.001), supporting H3. Time 2 intolerance of uncertainty was also positively associated with Time 2 future-oriented repetitive negative thinking (*β* = 0.40, *SE* = 0.03, 95% CI [0.34, 0.46], *p <* 0.001), supporting H5. Furthermore, Time 2 future-oriented repetitive negative thinking was positively associated with Time 3 employment anxiety (*β* = 0.19, *SE* = 0.03, 95% CI [0.13, 0.25], *p <* 0.001), supporting H6. These coefficients represent conditional associations within the specified structural model and should not be interpreted as estimates of causal effects.

Baseline employment anxiety showed a strong positive association with Time 3 employment anxiety (*β* = 0.46, *SE* = 0.03, 95% CI [0.40, 0.52], *p <* 0.001), indicating substantial temporal stability in employment anxiety. Nevertheless, the key paths from academic stress to intolerance of uncertainty, from intolerance of uncertainty to future-oriented repetitive negative thinking, and from future-oriented repetitive negative thinking to subsequent employment anxiety remained significant. These results support the proposed cognitive-affective pathway from academic stress to employment anxiety. The bootstrap estimates of the three specific indirect effects, the total indirect effect, the direct effect, and the total effect are presented in **Table 7**.

**Table 7:** Bootstrap tests of indirect and sequential mediation effects.

Indirect pathway	Estimate	*SE*	95% Bootstrap CI	Effect proportion
Time 1 academic stress → Time 2 intolerance of uncertainty → Time 3 employment anxiety	0.029	0.010	[0.012, 0.051]	25.66%
Time 1 academic stress → Time 2 future-oriented RNT → Time 3 employment anxiety	0.015	0.007	[0.004, 0.031]	13.54%
Time 1 academic stress → Time 2 intolerance of uncertainty → Time 2 future-oriented RNT → Time 3 employment anxiety	0.018	0.006	[0.008, 0.034]	16.25%
Total indirect effect	0.062	0.015	[0.034, 0.096]	55.45%
Direct effect	0.050	0.021	[0.010, 0.090]	44.55%
Total effect	0.112	0.033	[0.046, 0.178]	100.00%

RNT, repetitive negative thinking. Indirect effects were tested using 5,000 bootstrap resamples. An indirect effect was considered significant when the 95% bootstrap confidence interval did not include zero. Effect proportions were calculated using unrounded estimates.

All demographic and job-search-related covariates were entered simultaneously into the Time 3 employment anxiety equation in the final structural model. As shown in **Supplementary Table 1**, educational level, internship experience, current job-search status, and perceived family employment expectation were significantly associated with Time 3 employment anxiety, whereas the other covariate associations were not statistically significant. Complete standardized coefficients, standard errors, 95% confidence intervals, and *p*-values are provided in **Supplementary Table 1**.

### 4.5 Robustness and multi-group analyses

Robustness and multi-group analyses were conducted to examine the stability of the sequential mediation model. First, the model was re-estimated using observed scale mean scores instead of latent variables. The sequential indirect effect remained significant, with an estimate of 0.014 and a 95% bootstrap confidence interval of [0.006, 0.027]. Second, the model was re-estimated without controlling for baseline employment anxiety. The sequential indirect effect was also significant, with an estimate of 0.024 and a 95% bootstrap confidence interval of [0.011, 0.043]. These results indicated that the proposed cognitive-affective pathway was robust across alternative model specifications. The two robustness analyses and the summary results of the multi-group comparisons are presented in **Table 8**.

**Table 8:** Robustness and multi-group analyses of the sequential mediation model.

Analysis	Key pathway/effect	Estimate	95% CI/*p*-value	Conclusion
Observed-score model	Sequential indirect effect	0.014	[0.006, 0.027]	Supported
Without baseline employment anxiety	Sequential indirect effect	0.024	[0.011, 0.043]	Supported
Gender multi-group analysis	δ*χ*^2^ test (δ*df* = 6)	8.43	*p* = 0.208	No significant heterogeneity
Educational-level multi-group analysis	δ*χ*^2^ test (δ*df* = 6)	9.15	*p* = 0.165	No significant heterogeneity

Note. Sequential indirect effect refers to the pathway from Time 1 academic stress to Time 3 employment anxiety through Time 2 intolerance of uncertainty and Time 2 future-oriented repetitive negative thinking. δ*df* = 6 reflects the simultaneous constraint of the six primary structural paths across groups. Baseline employment anxiety and demographic covariates were included in the primary robustness and multi-group models.

Before comparing the structural paths, progressive measurement-invariance analyses were conducted using the parcel-based four-factor measurement model. For gender, the configural model showed good fit, with *χ*^2^ = 342.518, *df* = 150, CFI = 0.972, TLI = 0.966, RMSEA = 0.034, and SRMR = 0.030. Imposing equality constraints on the factor loadings resulted in only minimal changes in fit (|δCFI| = 0.001, δRMSEA = 0.001, and δSRMR = 0.003). Further constraining the indicator intercepts also produced changes below the prespecified thresholds (|δCFI| = 0.002, δRMSEA = 0.002, and δSRMR = 0.004). These results supported configural, metric, and scalar invariance across male and female participants. For educational level, the configural model also showed acceptable fit, with *χ*^2^ = 351.624, *df* = 150, CFI = 0.968, TLI = 0.962, RMSEA = 0.036, and SRMR = 0.032. The changes associated with the metric model were |δCFI| = 0.001, δRMSEA = 0.001, and δSRMR = 0.004. The corresponding changes for the scalar model were |δCFI| = 0.003, δRMSEA = 0.002, and δSRMR = 0.004. Thus, configural, metric, and scalar invariance were also supported across undergraduate and master’s students. Following the establishment of measurement invariance, the six primary structural paths were compared across groups. The path-equality constrained model did not fit significantly worse than the unconstrained structural model for either gender (δ*χ*^2^ = 8.432, δ*df* = 6, *p* = 0.208) or educational level (δ*χ*^2^ = 9.153, δ*df* = 6, *p* = 0.165). Therefore, no significant heterogeneity in the six primary structural paths was detected across male and female participants or across undergraduate and master’s students. Detailed measurement-invariance and structural-comparison results are presented in **Table 9**.

**Table 9:** Progressive measurement invariance and structural path comparisons across groups.

Grouping and Model	χ²	*df*	χ²/*df*	CFI	TLI	RMSEA	SRMR	ΔCFI	ΔRMSEA	ΔSRMR
Gender^a^										
Configural invariance	342.518	150	2.283	0.972	0.966	0.034	0.030	—	—	—
Metric invariance	354.102	160	2.213	0.971	0.967	0.033	0.033	0.001	0.001	0.003
Scalar invariance	371.846	170	2.187	0.969	0.966	0.035	0.037	0.002	0.002	0.004
Structural path comparison^d^	Δχ² = 8.432, Δ*df* = 6, *p* = 0.208 (No significant path heterogeneity)									
Educational level^b^										
Configural invariance	351.624	150	2.344	0.968	0.962	0.036	0.032	—	—	—
Metric invariance	365.281	160	2.283	0.967	0.963	0.035	0.036	0.001	0.001	0.004
Scalar invariance	383.095	170	2.254	0.964	0.960	0.037	0.040	0.003	0.002	0.004
Structural path comparison^d^	Δχ² = 9.153, Δ*df* = 6, *p* = 0.165 (No significant path heterogeneity)									

Absolute changes (δ) in fit indices are reported relative to the preceding measurement-invariance model. Configural invariance specified the same four-factor structure across groups. Metric invariance additionally constrained the factor loadings to equality, and scalar invariance further constrained the indicator intercepts to equality. *^a^* Male *n* = 452; female *n* = 593. Gender was excluded as a covariate from the gender-based structural path comparison models. *^b^* Undergraduate *n* = 891; master’s *n* = 154. Educational level was excluded as a covariate from the educational-level structural path comparison models. *^c^* The gender-based structural path comparison was conducted using a metric-invariant measurement model. The unconstrained structural model allowed the six primary structural paths to vary across groups, whereas the constrained model held these paths equal across groups.

*^d^* The educational-level structural path comparison was conducted using a metric-invariant measurement model. The unconstrained structural model allowed the six primary structural paths to vary across groups, whereas the constrained model held these paths equal across groups.

Fourth, an additional structural sensitivity model was estimated to examine whether the indirect pathways remained statistically significant when Time 1 baseline employment anxiety was associated with all subsequent endogenous variables. In this model, paths were specified from Time 1 baseline employment anxiety to Time 2 intolerance of uncertainty, Time 2 future-oriented repetitive negative thinking, and Time 3 employment anxiety. The sensitivity model showed good fit to the data: *χ*^2^ = 186.415, *df* = 82, *χ*^2^*/df* = 2.273, CFI = 0.968, TLI = 0.959, RMSEA = 0.035, and SRMR = 0.031. Time 1 baseline employment anxiety was positively associated with Time 2 intolerance of uncertainty (*β* = 0.104, *SE* = 0.031, 95% CI [0.043, 0.165], *p <* 0.001) and Time 2 future-oriented repetitive negative thinking (*β* = 0.142, *SE* = 0.029, 95% CI [0.085, 0.199], *p <* 0.001). After these paths were included, the associations of Time 1 academic stress with Time 2 intolerance of uncertainty (*β* = 0.212, *SE* = 0.032, 95% CI [0.149, 0.275], *p <* 0.001) and Time 2 future-oriented repetitive negative thinking (*β* = 0.062, *SE* = 0.028, 95% CI [0.007, 0.117], *p* = 0.027) remained statistically significant, although their magnitudes were modestly attenuated. Bootstrap analyses using 5,000 resamples indicated that the indirect effects through intolerance of uncertainty (estimate = 0.025, 95% bootstrap CI [0.010, 0.046]), through future-oriented repetitive negative thinking (estimate = 0.012, 95% bootstrap CI [0.002, 0.027]), and through the theoretically specified sequential pathway (estimate = 0.016, 95% bootstrap CI [0.007, 0.031]) remained statistically significant. The total indirect effect was 0.053 (95% bootstrap CI [0.028, 0.085]). These results indicate that the specified indirect associations were not fully accounted for by baseline employment anxiety; however, they do not rule out confounding by unmeasured general negative affectivity or related dispositions.

An additional covariate-extended model was estimated to examine the associations of baseline employment anxiety and social-background variables with the two Time 2 cognitive constructs. Time 1 baseline employment anxiety and perceived family employment expectation were positively associated with both intolerance of uncertainty and future-oriented repetitive negative thinking. More advanced job-search status was associated with lower future-oriented repetitive negative thinking. The remaining covariate associations were not statistically significant. Complete results are reported in **Supplementary Table 2**.

## 5 Discussion

### 5.1 Principal findings and theoretical implications

This study examined how academic stress was associated with employment anxiety among graduating students through a theoretically specified sequential cognitive-affective pathway. The results showed that academic stress was positively associated with subsequent employment anxiety, even after controlling for baseline employment anxiety and demographic covariates. The indirect effects through intolerance of uncertainty, through future-oriented repetitive negative thinking, and through the theoretically specified pathway involving both mediators were statistically significant. The latter effect was consistent with the proposed theoretical ordering from intolerance of uncertainty to future-oriented repetitive negative thinking. However, because both mediators were assessed at Time 2, the analysis did not establish that intolerance of uncertainty temporally preceded future-oriented repetitive negative thinking. The result should therefore be interpreted as support for a theoretically specified sequential indirect association rather than as evidence of a temporally verified sequence. These findings are consistent with prior evidence that academic stress is closely linked to students’ mental health risks (2) and extend recent research on the relationship between academic stress and employment anxiety (6).

Theoretically, the present study extends the literature by shifting attention from general protective resources to more specific cognitive-affective vulnerability processes that may be relevant to the association between academic stress and employment anxiety. Previous research has emphasized the role of resilience in this relationship (6). The present pattern of indirect associations suggests that uncertainty-related threat appraisal and perseverative future-oriented thinking may help characterize how academic stress and employment anxiety are related during the education-to-employment transition. This interpretation is consistent with evidence identifying intolerance of uncertainty as an important correlate of anxiety-related difficulties (9, 10) and repetitive negative thinking as a transdiagnostic process associated with emotional distress (13–15). However, the present findings do not establish that these variables operate as causal mechanisms, nor do they exclude stable psychological predispositions or reciprocal relationships. Employment anxiety among graduating students may therefore be understood as a mental health concern associated with academic stress, uncertainty-related cognitive vulnerability, and persistent future-oriented negative thinking, rather than solely as a career-planning problem.

The possible conceptual overlap between future-oriented repetitive negative thinking and employment anxiety should also be considered. Both constructs include negative anticipation of future employment-related threats, and the contextualized PTQ instructions may have increased the salience of employment concerns. Nevertheless, they differ in their primary conceptual focus. Future-oriented repetitive negative thinking refers to a cognitive process characterized by repetitiveness, intrusiveness, difficulty disengaging, perceived unproductiveness, and occupation of mental capacity (21). Its items assess how individuals engage with negative thoughts across academic completion, future employment, and post-graduation life. Employment anxiety, in contrast, is a domain-specific psychological response encompassing employment competition pressure, lack of employment support, lack of self-confidence, and concerns about employment prospects (27). Consistent with this distinction, future-oriented repetitive negative thinking and Time 3 employment anxiety were moderately correlated in the present study (*r* = 0.32), and the square roots of their average variance extracted values were approximately 0.75 and 0.72, respectively, both exceeding their interconstruct correlation. Together with the good fit of the four-factor measurement model, these results support their empirical distinguishability. However, discriminant-validity evidence does not completely eliminate possible shared content or shared method variance. The contextualized PTQ instructions and reliance on self-report measures may have contributed to the observed association. Future studies could use domain-general PTQ instructions, separate measures of employment-specific worry, and multimethod assessments to distinguish the cognitive process of repetitive negative thinking from the affective and evaluative experience of employment anxiety more rigorously.

### 5.2 Practical implications for university mental health and career support

The findings have implications for identifying students at elevated risk during the education-to-employment transition. University support systems often focus on observable employment outcomes, such as whether students have submitted applications, attended interviews, or received job offers. However, the present results suggest that psychological processes before and during the job-search period are also important. Students with high academic stress, high intolerance of uncertainty, and persistent future-oriented negative thinking may be particularly vulnerable to employment anxiety. This is consistent with broader evidence that college students face increasing mental health challenges (35) and that career-related anxiety can interfere with academic and career functioning (8).

The findings identify several potential points of attention for university support services, but they should not be interpreted as demonstrating the effectiveness of any specific intervention. Universities may consider integrating academic support, career guidance, and mental health services rather than treating them as entirely separate systems. Academic advising and thesis-management support may help students manage immediate academic demands, while career services may reduce avoidable uncertainty by providing clearer information about recruitment timelines, job-search strategies, and realistic career pathways. Psychological programs addressing intolerance of uncertainty and repetitive negative thinking may also warrant evaluation in graduating-student populations. Existing evidence suggests that scalable university mental health programs can reduce psychological distress (36, 37) and that interventions addressing worry, rumination, and repetitive negative thinking may benefit young people and university students (15, 38, 39). However, the intervention implications of the present findings remain provisional. Randomized or quasi-experimental studies are needed to determine whether modifying intolerance of uncertainty or repetitive negative thinking leads to reductions in employment anxiety.

The direct association between Time 1 academic stress and Time 3 employment anxiety was statistically significant but small (*β* = 0.050, 95% CI [0.010, 0.090]) after baseline employment anxiety and the other covariates were controlled. Although this direct component represented 44.55% of the total statistical association, its absolute magnitude indicated only a limited incremental association. Academic stress may therefore be understood as a relatively distal contextual correlate, while prior anxiety and other personal and job-search conditions remain important. Accordingly, the statistical significance of this path should not be interpreted as evidence of a large practical effect. The three specific indirect effects provide complementary rather than competing explanations of the association. The indirect effect through intolerance of uncertainty was 0.029 and accounted for 25.66% of the total association, whereas the indirect effect through future-oriented repetitive negative thinking was 0.015 and accounted for 13.54%. The theoretically ordered indirect effect involving both variables was 0.018 and accounted for a further 16.25%. Together, the indirect effects accounted for 55.45% of the total association, suggesting that uncertainty-related appraisal and repetitive negative thinking may jointly contribute to the association between academic stress and later employment anxiety. Although the indirect effect through intolerance of uncertainty was numerically larger, pairwise differences between the specific indirect effects were not formally tested; therefore, no conclusion about the relative dominance of one pathway is warranted.

### 5.3 Limitations and future research

Several limitations should be acknowledged. First, although the three-wave time-lagged design strengthens the temporal ordering of academic stress and subsequent employment anxiety compared with cross-sectional research, causal inference remains limited. Second, intolerance of uncertainty and future-oriented repetitive negative thinking were both measured at Time 2. Therefore, their ordering was specified according to the theoretical model rather than established through temporal separation. The significant sequential indirect effect was consistent with the proposed theoretical ordering, but it did not demonstrate that intolerance of uncertainty temporally preceded future-oriented repetitive negative thinking. Reciprocal or alternative ordering between the two constructs cannot be excluded. Third, all variables were measured using self-report questionnaires, which may introduce subjective reporting bias. Fourth, the sample was drawn from universities in Shaanxi Province, China, so the generalizability of the findings to other regions, institutional types, or cultural contexts should be examined in future studies. The generalizability of the findings should also be considered in relation to the study’s regional and institutional context.

The generalizability of the findings should also be considered in relation to the study’s regional and institutional context. Because the sample was recruited through non-probability convenience sampling from participating universities in Shaanxi Province, it should not be regarded as nationally representative of all graduating students in China. Students in other regions may encounter different labor-market conditions, recruitment opportunities, geographic mobility expectations, living costs, and university career-support systems, which could influence the strength or pattern of the observed associations. The findings also cannot be assumed to generalize to students in vocational colleges or other institutional settings not adequately represented in the present sample, students who are not approaching graduation, or graduates who have already entered the labor market. Future research should recruit participants from institutions across different regions of China, include a broader range of educational and institutional contexts, and use probability-based or stratified sampling where feasible. Such research could also examine whether regional employment conditions and institutional support systems modify the associations among academic stress, intolerance of uncertainty, repetitive negative thinking, and employment anxiety.

The sequential indirect effect requires particular caution in interpretation. Because intolerance of uncertainty and future-oriented repetitive negative thinking were both assessed at Time 2, the estimated pathway represents a theoretically specified statistical ordering rather than a temporally observed progression between the mediators. Reverse or reciprocal relations and unmeasured common antecedents cannot be excluded, and the absence of baseline measurements of the two mediators prevents an assessment of within-person changes in these constructs. Bootstrap confidence intervals quantify the statistical uncertainty of the indirect effect but do not establish its temporal or causal ordering. Future studies should measure the two mediators at separate waves and compare alternative directional models.

The study did not include a direct measure of general negative affectivity or related dispositional characteristics. Although controlling for Time 1 employment anxiety reduced the possibility that the findings merely reflected the stability of employment-specific anxiety, baseline employment anxiety cannot fully represent broader tendencies such as neuroticism, depressive disposition, pessimism, or a generally negative response style. Consequently, unmeasured dispositional factors may partly account for the observed associations. Future research should include direct measures of general negative affectivity and related personality and emotional characteristics to determine whether the observed associations are specific to the education-to-employment transition or reflect a broader psychological vulnerability.

The study also did not include a measure of a neutral or unbiased attitude against which the negative cognitive variables could be compared. Related positive or non-negative dispositions, such as optimism, positive affectivity, and a less negatively biased response style, were not assessed. Therefore, the available data cannot determine whether the observed associations are specific to negative cognitive vulnerability or partly reflect a broader response tendency. Future studies should include negative, positive, and neutral dispositional measures within the same design to provide a more balanced evaluation of these alternative explanations.

Future research can extend this work in several directions. A four-wave longitudinal design could measure academic stress, intolerance of uncertainty, future-oriented repetitive negative thinking, and employment anxiety at separate time points to test the proposed sequence more rigorously. Experience-sampling designs could further capture how daily academic demands and employment-related uncertainty trigger momentary worry and anxiety. Future studies may also incorporate objective employment indicators, such as number of job applications, interview outcomes, offer status, and duration of job search. Finally, intervention studies are needed to test whether reducing intolerance of uncertainty and repetitive negative thinking can lower employment anxiety among graduating students. Such work would help move the present model from explanation toward prevention and targeted mental health support.

## 6 Conclusion

This study investigated how academic stress was associated with employment anxiety among graduating students through a theoretically specified sequential cognitive-affective pathway. Using a three-wave time-lagged survey of 1,045 Chinese graduating students, the findings showed that academic stress was positively associated with subsequent employment anxiety, even after controlling for baseline employment anxiety and demographic covariates. Significant indirect effects were identified through intolerance of uncertainty, through future-oriented repetitive negative thinking, and through the theoretically specified sequential pathway involving both mediators. However, because intolerance of uncertainty and future-oriented repetitive negative thinking were both measured at Time 2, the findings do not establish temporal precedence between them. The significant sequential indirect effect should therefore be interpreted as being consistent with the proposed theoretical ordering rather than as evidence of a temporally verified sequence. The findings highlight the importance of integrating academic support, career guidance, and mental health interventions that help graduating students manage uncertainty and reduce unproductive future-oriented worry.

## Data Availability

The original contributions presented in the study are included in the article/**Supplementary Material**. Further inquiries can be directed to the corresponding author.

## Supplementary material

Supplementary Table 1 Standardized coefficients for control variables in the Time 3 employment anxiety equation *Note.* Standardized coefficients are reported. All covariates were entered simultaneously into the Time 3 employment anxiety equation in the final structural model. Model-based 95% confidence intervals are reported. ^a^ Reference group: Male. ^b^Reference group: Undergraduate. ^c^ Reference group: Rural. ^d^ Reference group: Non-only-child. ^e^ Reference group: No internship experience. ^f^ Treated as an ordinal trend variable coded as 0 = not yet started, 1 = preparing, 2 = actively applying, and 3 = received at least one offer. ^g^ Reference group: Below 5,000 RMB. ^h^ Reference group: Humanities and Social Sciences.

Supplementary Table 2 Standardized associations of baseline employment anxiety and covariates with Time 2 cognitive constructs *Note.* Standardized coefficients are reported. Model-based 95% confidence intervals are presented. The estimates were obtained from an additional covariate-extended model in which Time 1 baseline employment anxiety and all listed covariates were entered simultaneously into the equations for both Time 2 constructs. This model was separate from the structural sensitivity model reported in the main Results section. *^a^* Reference group: Male. *^b^* Reference group: Undergraduate. *^c^* Reference group: Rural. *^d^* Reference group: Non-only-child. *^e^* Reference group: No internship experience. *^f^* Treated as an ordinal trend variable coded as 0 = not yet started, 1 = preparing, 2 = actively applying, and 3 = received at least one offer. *^g^* Reference group: Below 5,000 RMB. *^h^* Reference group: Humanities and Social Sciences. RNT = repetitive negative thinking. Future-oriented RNT was examined as the closest available measure of future-oriented negative cognition; it was not a direct measure of negative expectations or a general negative psychological disposition.
