# Supplementary material for: Academic stress and employment anxiety among graduating students: intolerance of uncertainty and future-oriented repetitive negative thinking as sequential cognitive-affective pathways
Source: Front Psychiatry. 2026 Aug 19;17:1897109. doi: 10.3389/fpsyt.2026.1897109 (PMC13533917; doi:10.3389/fpsyt.2026.1897109)
Supplement: Supplementary file 1 [file Table1.pdf]

Supplementary Material

Academic Stress and Employment Anxiety Among Graduating Students: Intolerance of Uncertainty and Future-Oriented Repetitive Negative Thinking as Sequential Cognitive-Affective Pathways

Mei Zhou and Na Ni

Table S1. Standardized coefficients for control variables in the Time 3 employment anxiety equation

Covariate	β	SE	95% CI	p-value
Gender (Female)[a]	0.045	0.029	[−0.012, 0.102]	0.124
Age	−0.016	0.023	[−0.061, 0.029]	0.482
Educational Level (Master's)[b]	0.069	0.029	[0.012, 0.126]	0.018
Household Registration (Urban)[c]	−0.032	0.029	[−0.089, 0.025]	0.265
Only-Child Status (Yes)[d]	0.051	0.029	[−0.006, 0.108]	0.081
Internship Experience (Yes)[e]	−0.084	0.028	[−0.139, −0.029]	0.003
Current Job-Search Status[f]	−0.129	0.023	[−0.174, −0.084]	< 0.001
Perceived Family Employment Expectation	0.112	0.029	[0.055, 0.169]	< 0.001
Monthly Household Income[g]				
5,000–10,000 RMB	−0.021	0.024	[−0.068, 0.026]	0.381
Above 10,000 RMB	−0.043	0.031	[−0.104, 0.018]	0.166
Academic Discipline[h]				
Economics and Management	0.012	0.031	[−0.049, 0.073]	0.698
Science and Engineering	−0.024	0.028	[−0.079, 0.031]	0.392
Education	0.005	0.033	[−0.060, 0.070]	0.879
Medical and Health-Related	0.031	0.035	[−0.038, 0.100]	0.377

Note. Standardized coefficients are reported. All covariates were entered simultaneously into the Time 3 employment anxiety equation in the final structural model. Model-based 95% confidence intervals are reported.

[a] Reference group: Male.
[b] Reference group: Undergraduate.
[c] Reference group: Rural.
[d] Reference group: Non-only-child.
[e] Reference group: No internship experience.
[f] Treated as an ordinal trend variable coded as 0 = not yet started, 1 = preparing, 2 = actively applying, and 3 = received at least one offer.
[g] Reference group: Below 5,000 RMB.
[h] Reference group: Humanities and Social Sciences.

Table S2. Standardized associations of baseline employment anxiety and covariates with Time 2 cognitive constructs

Covariate	β	SE	95% CI	p-value
**Panel A. Associations with Time 2 Intolerance of Uncertainty**				
Time 1 Baseline Employment Anxiety	0.095	0.029	[0.038, 0.152]	0.001
Gender (Female)[a]	0.031	0.028	[−0.024, 0.086]	0.268
Age	0.014	0.024	[−0.033, 0.063]	0.559
Educational Level (Master's)[b]	0.052	0.029	[−0.005, 0.109]	0.074
Household Registration (Urban)[c]	−0.016	0.029	[−0.073, 0.041]	0.584
Only-Child Status (Yes)[d]	0.036	0.029	[−0.021, 0.093]	0.215
Internship Experience (Yes)[e]	−0.042	0.028	[−0.097, 0.013]	0.134
Current Job-Search Status[f]	−0.035	0.021	[−0.076, 0.004]	0.095
Perceived Family Employment Expectation	0.081	0.029	[0.024, 0.138]	0.005
Monthly Household Income[g]				
5,000–10,000 RMB	−0.024	0.024	[−0.071, 0.023]	0.318
Above 10,000 RMB	−0.039	0.031	[−0.100, 0.022]	0.209
Academic Discipline[h]				
Economics and Management	0.015	0.031	[−0.046, 0.076]	0.628
Science and Engineering	−0.019	0.028	[−0.074, 0.036]	0.498
Education	0.008	0.033	[−0.057, 0.073]	0.807
Medical and Health-Related	0.026	0.035	[−0.043, 0.090]	0.457
**Panel B. Associations with Time 2 Future-Oriented RNT**				
Time 1 Baseline Employment Anxiety	0.148	0.028	[0.093, 0.203]	< 0.001
Gender (Female)[a]	0.048	0.029	[−0.009, 0.105]	0.098
Age	−0.011	0.023	[−0.056, 0.034]	0.634
Educational Level (Master's)[b]	0.031	0.029	[−0.026, 0.088]	0.287
Household Registration (Urban)[c]	−0.007	0.029	[−0.064, 0.050]	0.810
Only-Child Status (Yes)[d]	0.016	0.029	[−0.041, 0.073]	0.582
Internship Experience (Yes)[e]	−0.032	0.028	[−0.087, 0.023]	0.252
Current Job-Search Status[f]	−0.061	0.021	[−0.102, −0.020]	0.004
Perceived Family Employment Expectation	0.068	0.029	[0.011, 0.125]	0.017
Monthly Household Income[g]				
5,000–10,000 RMB	−0.016	0.024	[−0.063, 0.031]	0.504
Above 10,000 RMB	−0.028	0.031	[−0.089, 0.033]	0.369
Academic Discipline[h]				
Economics and Management	0.008	0.031	[−0.053, 0.069]	0.796
Science and Engineering	−0.014	0.028	[−0.069, 0.041]	0.617
Education	0.002	0.033	[−0.063, 0.067]	0.952
Medical and Health-Related	0.021	0.035	[−0.048, 0.090]	0.548

Note. Standardized coefficients are reported. Model-based 95% confidence intervals are presented. The estimates were obtained from an additional covariate-extended model in which Time 1 baseline employment anxiety and all listed covariates were entered simultaneously into the equations for both Time 2 constructs. This model was separate from the structural sensitivity model reported in the main Results section.

[a] Reference group: Male. [b] Reference group: Undergraduate. [c] Reference group: Rural. [d] Reference group: Non-only-child. [e] Reference group: No internship experience.

[f] Treated as an ordinal trend variable coded as 0 = not yet started, 1 = preparing, 2 = actively applying, and 3 = received at least one offer.

[g] Reference group: Below 5,000 RMB. [h] Reference group: Humanities and Social Sciences.

RNT = repetitive negative thinking. Future-oriented RNT was examined as the closest available measure of future-oriented negative cognition; it was not a direct measure of negative expectations or a general negative psychological disposition.
